# MGCNSS: miRNA–disease association prediction with multi-layer graph convolution and distance-based negative sample selection strategy

**DOI:** 10.1093/bib/bbae168

**Published:** 2024-04-15

**Authors:** Zhen Tian, Chenguang Han, Lewen Xu, Zhixia Teng, Wei Song

**Affiliations:** School of Computer and Artificial Intelligence, Zhengzhou University, Zhengzhou 450000, China; Yangtze Delta Region Institute (Quzhou), University of Electronic Science and Technology of China, Quzhou 324000, China; School of Computer and Artificial Intelligence, Zhengzhou University, Zhengzhou 450000, China; School of Computer and Artificial Intelligence, Zhengzhou University, Zhengzhou 450000, China; College of Computer and Control Engineering, Northeast Forestry University, Harbin 150040, China; School of Computer and Artificial Intelligence, Zhengzhou University, Zhengzhou 450000, China

**Keywords:** graph convolutional network, meta-path, distance-based negative sample selection, miRNA–disease association

## Abstract

Identifying disease-associated microRNAs (miRNAs) could help understand the deep mechanism of diseases, which promotes the development of new medicine. Recently, network-based approaches have been widely proposed for inferring the potential associations between miRNAs and diseases. However, these approaches ignore the importance of different relations in meta-paths when learning the embeddings of miRNAs and diseases. Besides, they pay little attention to screening out reliable negative samples which is crucial for improving the prediction accuracy. In this study, we propose a novel approach named MGCNSS with the multi-layer graph convolution and high-quality negative sample selection strategy. Specifically, MGCNSS first constructs a comprehensive heterogeneous network by integrating miRNA and disease similarity networks coupled with their known association relationships. Then, we employ the multi-layer graph convolution to automatically capture the meta-path relations with different lengths in the heterogeneous network and learn the discriminative representations of miRNAs and diseases. After that, MGCNSS establishes a highly reliable negative sample set from the unlabeled sample set with the negative distance-based sample selection strategy. Finally, we train MGCNSS under an unsupervised learning manner and predict the potential associations between miRNAs and diseases. The experimental results fully demonstrate that MGCNSS outperforms all baseline methods on both balanced and imbalanced datasets. More importantly, we conduct case studies on colon neoplasms and esophageal neoplasms, further confirming the ability of MGCNSS to detect potential candidate miRNAs. The source code is publicly available on GitHub https://github.com/15136943622/MGCNSS/tree/master

## INTRODUCTION

MicroRNA (miRNA) is one type of non-coding single-stranded-RNAs, which usually has a length of about 22 nucleotides [1]. Research has demonstrated that many miRNAs could participate in the regulation of gene expression after transcription in both animals and plants [2]. They could also serve as potential diagnostic markers and therapeutic targets [3]. These miRNAs regulate about one-third of human genes that are highly associated with complex human diseases. For example, miR-17, miR-92a and miR-31 have been validated as biomarkers for colorectal cancer, which is crucial for the diagnosis and treatment of colorectal cancer [4]. Thus, identifying the associations between miRNAs and diseases could promote research on the mechanism of diseases and help the treatment of diseases.

Identifying miRNA–disease associations through the wet-experimental strategy is time-consuming, labor-intensive and low efficiency [5]. More recently, the development of biotechnology has promoted the emergence of computational-based methods which could greatly improve prediction efficiency. Currently, these computational-based approaches can be divided into three categories: similarity-based methods, machine learning-based methods and graph-based methods [6].

For these similarity-based methods, they usually pay much attention to the similarities calculation between miRNAs and diseases. Therefore, miRNAs and diseases will represented as feature vectors utilizing their multiple types of biological data, such as miRNA sequence, miRNA annotation and gene annotations. The similarity-based methods generally assume that functional similarity miRNAs tend to be associated with these diseases that share similar phenotypes [7]. Up to now, various similarity calculation models such as disease semantic similarity [8, 9], miRNA functional similarity [10] and Gaussian Interaction Profile (GIP) kernel similarity [11] have been proposed to measure their similarity effectively. For example, Jiang [12] put forward a novel kernel-based fusion strategy to integrate multiple similarities of miRNAs and diseases, and then predict their potential association relationships. GATMDA [13] treated lncRNAs as mediators and put forward the lncRNA–miRNA–disease regulatory mechanism to enhance the similarity calculation of miRNAs and diseases. Besides, Yu *et al*. [14] employed the miRNA target genes with GO annotations to systematically measure the similarity between miRNAs. To fully use the global network similarity measures, Chen et al. [15] employed the Random Walk with Restart (RWR) algorithm on the miRNA-miRNA functional similarity network to predict miRNA–disease association. MFSP [16] inferred the functional similarity of miRNAs with the pathway-based miRNA-miRNA relations and measured the miRNS similarities between their corresponding associated disease sets. Mørk et al. [17]. constructed one miRNA-target-disease heterogeneous network and treated proteins as the intermediary to measure the similarities between miRNAs and diseases. Although these approaches above could measure the similarity between miRNAs or diseases, their calculation models are relatively simple. More importantly, they pay little attention to utilizing the complex relationships of miRNAs and diseases in the heterogeneous network, which could limit their accuracy in the similarity calculation.

Machine learning-based approaches usually employ various efficient models such as regularized learning, Random Walk, Support Vector Machine (SVM [18]), or decision trees to discover potential miRNA–disease associations. These methods usually have two steps, which are the training step and the inference step [19]. For example, LRLSHMDA [20] was a semi-supervised model that employed a Laplacian regularized least squares classifier and effectively utilized the implicit information of vertices and edges to infer microbe-disease associations. AMVML [21] was an adaptive learning-based approach, which could learn novel similarity graphs and similarity graphs for miRNAs and diseases from different views and predict the miRNA–disease associations. EDTMDA [22] put forward one computational framework that adopted the dimensionality reduction strategy to remove redundant features and employed multiple decision trees to infer the association relationships. MTLMDA [23] employed the multi-task learning technique which could exploit both miRNA–disease and gene-disease networks at the same time to complete the association prediction task. EGBMMDA [24] trained a regression tree in a gradient boosting framework, which was the first decision tree-based model used to predict miRNA–disease associations. RFMDA [25] selected robust features with the filter-based feature selection strategy and adopted the Random Forest (RF) model as a classifier to infer the association relationship between miRNAs and diseases. Meanwhile, DNRLMF-MDA [11] calculated association probabilities through logical matrix factorization and further improved predictive performance through dynamic neighborhood regularization. MTDN learned the features from the DTINET [26] adopted the RWR algorithm to obtain initial features and compressed features with DCA and applied the matrix completion approach to calculate projections between different nodes. CCL-DTI [27] treated the multimodal knowledge as input and investigated the effectiveness of the different contrastive loss on the prediction model. At the same time, DeepCompoundNet [28] fully considered multi-source of chemicals and proteins such as protein features and drug properties to predict drug–target interactions. However, these above models mainly have two drawbacks: (1) they usually concatenated the embeddings of miRNAs and diseases learned from different sources, and (2) they failed to extract discriminative embeddings of miRNAs and diseases from the networks with rich semantic information, which limits the ability in learning the representations.

Meanwhile, Graph-based methods have attracted more and more attention due to their outstanding performance. These approaches always construct one heterogeneous graph based on the known miRNA–disease association relationships as well as the similarities of miRNAs and diseases [29]. The heterogeneous graph exhibits a powerful ability to depict the complex relationships between miRNAs and diseases, which has been commonly employed in miRNA–disease association prediction tasks. For example, GCN and GAT models were widely adopted in the field of graph learning due to their powerful performance [30]. Recently, MMGCN [31] utilized GCN as encoders to obtain features of miRNAs and diseases under different similarity views and then enhanced the representation learning process through multi-channel attention mechanisms. GCNDTI [32] constructed a drug-protein pair network and treated node pairs as independent nodes, which transformed the link prediction problem into a node pair classification problem. MAGCN [33] introduced lncRNA-miRNA interactions and miRNA–disease associations to represent miRNAs and diseases, then did MDA predictions via graph convolution networks with the multi-channel attention mechanism and convolutional neural network combiner. MKGAT [34] applied multi-layer GAT to update miRNA or disease features and then fused them through the attention mechanism. AMHMDA [35] first constructed a heterogeneous hyper-graph and applied the attention-aware multi-view similarity strategy to learn the embeddings of miRNAs and diseases in the constructed hyper-graph. DTIHNC [36] utilized GAT and RWR models respectively to learn both direct neighbor and multi-hop neighbor information and then applied a multi-level CNN to fuse node features. HGIMDA [37] adopted a graph neural network-based encoder to aggregate node neighborhood information to obtain low-dimensional embeddings of nodes for association prediction. SFAGE [6] optimized the original features through random walks and added a reinforcement layer in the hidden layer of the graph convolutional network to preserve the similarity information in the feature space. AutoEdge-CCP [38] models circRNA-cancer-drug interactions by employing a multi-source heterogeneous network, where each molecule combines intrinsic attribute information. However, these approaches do not pay much attention to the multiple meta-path-based relationships between miRNAs and diseases, while these paths usually contain rich meaningful semantics that crucially contribute to the learning of miRNA and disease representations. Besides, they only randomly select the negative samples from the unlabeled sample set, which affects their training performance.

Generally, heterogeneous networks face certain difficulties in the representation learning of nodes due to their complicated structure [39]. Hence, is one of the great challenges to design an automated learning framework to fully explore the complex meta-path-based relationships over heterogeneous graphs for embedding learning [40]. In addition, it is difficult to obtain high-quality negative samples for the miRNA–disease association prediction task. Consequently, lots of current approaches usually select samples from unlabeled sample sets as negative samples randomly, but there often exist numerous false negative samples, thus affecting the accuracy of prediction models. Therefore, selecting high-reliable and quality negative training samples is of great significance for miRNA–disease association prediction tasks.

**Figure 1:**
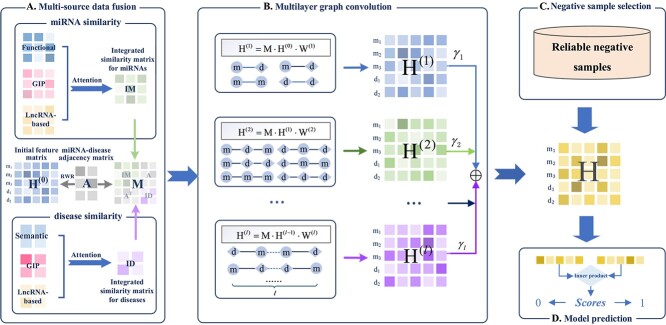
The overall architecture of MGCNSS, which mainly has four steps. (A) MGCNSS establishes the integrated miRNA and disease similarity matrix by fusing their different types of similarities. Then, the RWR algorithm is applied to learn the initial feature matrix ${H^{(0)}}$ for miRNAs and diseases. (B) MGCNSS adopts a multi-layer graph convolution module to capture the rich semantics of meta-paths with different lengths and learns the discriminative embeddings of miRNAs and diseases. (C) MGCNSS employs cosine-distance and Euclidean-distance-based strategies to select high-quality negative samples. (D) MGCNSS predicts the miRNA–disease associations with their learned embeddings.

In summary, we propose a novel prediction model with the multi-layer graph convolution and negative sample selection strategy (named MGCNSS). Firstly, we collect their multi-types of similarity and obtain the integrated miRNA and disease similarity networks to fully capture the similarity between miRNAs or diseases. Then, we adopt the multi-layer graph convolution module to automatically capture the rich semantics of meta-paths with lengths between miRNAs and diseases and learn their embeddings from different layers. After that, we adopt the negative sample selection strategy to screen out the high-quality negative training samples with the distanced-based strategy. Finally, MGCNSS predicts the miRNA–disease associations with the learned embeddings of miRNAs and diseases. The workflow has been displayed in Figure 1. We summarize the contributions of this study as follows:

- MGCNSS could automatically capture the rich semantics of meta-path with different lengths between miRNAs and diseases for learning their discriminative embeddings.
- A negative sample selection strategy is put forward to screen out high-reliable negative samples for training, which could enhance the performance of the prediction model.
- We conduct comprehensive experiments on balanced and unbalanced datasets, and the results demonstrate that MGCNSS outperforms all baseline methods on the evaluation metrics.

## MATERIALS AND METHODS

### Dataset

In this study, we collected the experimental data from HMDD v2.0 [41], which has 495 miRNAs, 383 diseases, and 5430 miRNA–disease associations that have been experimentally verified. Further, MGCNSS could construct the miRNA–disease association network and the corresponding association matrix that is denoted as $A=[A_{ij}]\in \mathbb{R}^{N_{m}\times N_{d}}$, where $N_{m}$ and $N_{d}$ represent the number of miRNAs and diseases, respectively. For matrix$A$, if $A_{ij}=1$, there will be an association relationship between $m_{i}$ and $d_{j}$. Otherwise, $A_{ij}=0$ indicates that there is no association relationship between $m_{i}$ and $d_{j}$.

### MiRNA functional similarity

We downloaded the miRNA functional similarity scores from HMDD [42]. In this dataset, the functional similarities between all miRNAs are represented in one matrix $FM\in \mathbb{R}^{N_{m}\times N_{m}}$ and $FM(m_{i},m_{j})$ denotes the similarity value between miRNA $m_{i}$ and miRNA $m_{j}$. It is worth noting that a higher level of similarity between two miRNAs if the corresponding score is higher.

### Disease semantic similarity

The relationships between diseases have been well described based on the Medical Subject Headings (MeSH) descriptors [43], which could be constructed as one directed acyclic graph (DAG). MGCNSS measures the disease semantic similarity based on this DAG. Specifically, MGCNSS calculates the semantic contribution of disease $d$ to $D$ according to the following formula: 


(1) \begin{align*}& \scriptsize DM1_{D}(d)= \left\{ \begin{aligned} &1,& if\;d =D\\ max\{\Delta\!\ast DM1_{D}(d^{{\prime}})& \lvert d^{{\prime}}\!\!\in\!children\;of\;d\},&if\;d\neq D \end{aligned}\right.,\end{align*}

where $d$ is the ancestor node of $D$, and $\Delta $ represents the semantic contribution decay factor, which is usually 0.5. According to equation (1), the semantic value of $D$ can be obtained by 


(2) \begin{align*}& DSM1(D)=\sum_{d\in T(D)}\!\!DM1_{D}(d),\end{align*}

where $T(D)$ denotes the ancestor node set of $D$ including $D$ itself. Therefore, the semantic similarity between disease $d_{i}$ and disease $d_{j}$ can be calculated as follows: 


(3) \begin{align*}& FD1(d_{i},d_{j})=\frac{\sum_{d_{r}\in T(d_{i})\bigcap T(d_{j})}\!\big(DM1_{d_{i}}(d_{r})\!+\!DM1_{d_{j}}(d_{r})\big)}{DSM1(d_{i})+DSM1(d_{j})}\end{align*}

Besides, if disease $d$ occurs only in the DAG of one disease $D$, but not in the DAG of other diseases, it is necessary to increase the contribution score of disease $d$ to $D$ [44]. Therefore, MGCNSS measures the semantic contribution of disease $d$ to $D$, which is formulated as follows: 


(4) \begin{align*}& DM2_{D}(d)=-log{\left(\frac{the\;number\;of\;DAGs\;including\;d}{the\;total \:number\;of\;diseases}\right)}\end{align*}

Similar with the calculation strategy of $FD1$, we can formulate the equation for $DSM2$ and $FD2$: 


(5) \begin{align*} & DSM2(D)=\sum_{d\in T(D)}DM2_{D}(d), \end{align*}

(6) \begin{align*} & FD2(d_{i},d_{j})=\frac{\sum_{d_{r}\in T(d_{i})\bigcap T(d_{j})}\!\big(DM2_{d_{i}}(d_{r})\!+\!DM2_{d_{j}}(d_{r})\big)}{DSM2(d_{i})+DSM2(d_{j})}.\end{align*}

Finally, these two types of calculation approaches are combined: 


(7) \begin{align*}& FD={(FD1+FD2)}\big/{2},\end{align*}

where $FD$ is the disease semantic similarity matrix.

### GIP kernel similarity

Similar to the previous research [45], MGCNSS measures the $GIP$ kernel similarity [11] for miRNAs and diseases based on the miRNA–disease adjacency matrix $A$.

Take building the $GIP$ kernel similarity for miRNA $(GM)$ as an example. Firstly, in the term of the adjacency matrix $A$, the $i$th and $j$th row are treated as the disease interaction profiles for miRNA $m_{i}$ and $m_{j}$, which are denoted as $R_{i}$ and $R_{j}$ [10]. Then, we measure the $GIP$ kernel similarity between miRNA $m_{i}$ and miRNA $m_{j}$ based on $R_{i}$ and $R_{j}$. The corresponding similarity could be constructed by 


(8) \begin{align*}& GM(m_{i},m_{j})=\text{exp}\left(-\alpha_{m}{\Vert R_{i} - R_{j}\Vert}^{2}\right),\end{align*}

where $\alpha _{m}$ is the controller of the bandwidth of the kernel and it can be calculated as follows: 


(9) \begin{align*}& \alpha_{m}=\alpha_{m}^{{\prime}}\bigg/\left(\frac{1}{N_{m}}\sum_{i=1}^{N_{m}}{\Vert R_{i}\Vert}^{2} \right),\end{align*}

where $N_{m}$ represents the number of miRNAs, and $\alpha _{m}^{{\prime}}$ is usually set to 1 referring to previous study.

In a similar manner, MGCNSS could establish the similarity matrix $GD$ for diseases. Specifically, MGCNSS treats columns in matrix $A$ as miRNA interaction profiles for the corresponding diseases. Without loss of generality, the relationship vectors for $d_{i}$ and $d_{j}$ are represented as $C_{i}$ and $C_{j}$, and their similarity is formulated as follows: 


(10) \begin{align*} & GD(d_{i},d_{j})=\text{exp}\left(-\beta_{m}{\Vert C_{i} - C_{j}\Vert}^{2}\right), \end{align*}

(11) \begin{align*} & \beta_{m}=\beta_{m}^{{\prime}}\bigg/\left(\frac{1}{N_{d}}\sum_{i=1}^{N_{d}}{\Vert C_{i}\Vert}^{2} \right),\end{align*}

where $N_{d}$ represents the number of diseases and $\beta _{m}^{{\prime}}$ is also set to 1.

### lncRNA-based similarity

Many studies have shown that lncRNAs participate in various biological processes, including DNA methylation, post-transcriptional regulation of RNA, and protein translation regulation. As a result, lncRNAs have association relationships with miRNAs and diseases. MGCNSS adopts the association relationships to measure the similarity between miRNAs and diseases separately.

The raw data is downloaded from Star-base v2.0 database [46], and the miRNA-lncRNA association matrix and disease-lncRNA association matrix can be obtained from GATMDA [13]. Finally, we adopt an edit-distance algorithm [47] to obtain the lncRNA-based similarity matrices for miRNAs and diseases, named $LM\in \mathbb{R}^{N_{m}\times N_{m}}$ and $LD\in \mathbb{R}^{N_{d}\times N_{d}}$, respectively.

### MGCNSS model

The overall architecture of MGCNSS has been demonstrated in Figure 1. The model architecture could be summarized into four main parts: multi-source data fusion, multi-layer graph convolution, negative sample selection and model prediction.

#### Multi-source data fusion

Now we have obtained miRNA functional similarity matrix($FM$), miRNA Gaussian kernel-based similarity matrix $GM$, and lncRNA-based miRNA similarity matrix $LM$, respectively. Next, MGCNSS could get an integrated miRNA similarity matrix name $IM$ based on $FM$, $GM$ and $LM$, which is formulated as follows: 


(12) \begin{align*}& IM=(\alpha_{1}\cdot FM + \alpha_{2}\cdot GM +\alpha_{3}\cdot LM)\end{align*}

where $\alpha _{1}$, $\alpha _{2}$ and $\alpha _{3}$ are hyper-parameters.

MGCNSS establishes the disease semantic similarity matrix $FD$, Gaussian kernel-based disease similarity matrix $GD$ and the lncRNA-based similarity disease matrix $LD$. The integrated disease similarity matrix $ID$ is denoted as follows: 


(13) \begin{align*}& ID=(\beta_{1}\cdot FD + \beta_{2}\cdot GD +\beta_{3}\cdot LD)\end{align*}

where $\beta _{1}$, $\beta _{2}$ and $\beta _{3}$ are hyper-parameters. These hyper-parameters are investigated in the Result section.

MGCNSS will construct the heterogeneous miRNA–disease association network $N_{hete}$, which is used for the multi-layer graph convolution module. The matrix representation of $N_{hete}$ is denoted as $M$, which is formulated as follows: 


(14) \begin{align*}&M= \begin{bmatrix}IM & A \\ A^{T} & ID \end{bmatrix}\end{align*}

where $IM\in \mathbb{R}^{N_{m}\times N_{m}}$, $ID\in \mathbb{R}^{N_{d}\times N_{d}}$ and $A\in \mathbb{R}^{N_{m}\times N_{d}}, M\in \mathbb{R}^{(N_{d}+N_{m})\times (N_{d}+N_{m})}$.

Finally, MGCNSS initializes the features of miRNAs and diseases with RWR algorithm [36] based on adjacency matrix $A$. Specifically, The RWR algorithm is formulated as follows: 


(15) \begin{align*}& D_{t}^{(k1)}=(1-r )A_{N} D_{t}^{(k-1)}+r D_{t}^{0},\end{align*}

where $r$ represents the restart probability and $t$ is the number of iterations. $D_{t}^{0}\in \mathbb{R}^{(N_{d}+N_{m})\times 1}$ is the initial vector of the $t$th node. Besides, $A_{N}$ is the normalized adjacency matrix $A$. The algorithm will be performed until $Frobenius |D^{(k+1)}-D^{k}| \leqslant 10^{-6}$ and $D_{t}^{k}$ is the out feature of the $t$th node at $k$th iteration. In this way, all the initial features of miRNAs and diseases are obtained and form the feature matrix denoted as $D^{k}$.

#### Multilayer graph convolution

Generally, meta-paths have a powerful ability to capture multiple relationships of nodes in the heterogeneous network. It is essential to comprehensively explore the rich complex semantics to learn the embeddings of miRNAs and diseases. Here we employ multi-layer graph convolution to learn their embeddings.

As shown in Figure 1(B), the graph convolutional module consists of multiple graph convolutional layers, which could fully capture the meaning of meta-paths with different lengths. Specifically, the first layer of convolution can be represented as follows: 


(16) \begin{align*}& H^{(1)}=M\cdot H^{(0)}\cdot W^{(1)},\end{align*}

where $H^{(1)}\in \mathbb{R}^{(N_{m}+N_{d})\times d}$ is the feature matrix for meta-paths with length 1. $H^{(0)}=D^{k}$ is the input feature that obtained from RWR, and $W^{(1)}\in \mathbb{R}^{(N_{m}+N_{d})\times d}$ is the learnable weight matrix, where $d$ is the embedding size for output features of miRNAs and diseases.

The two-layer convolution is formulated as follows: 


(17) \begin{align*}& \begin{aligned} H^{(2)} = & \ M\cdot H^{(1)}\cdot W^{(2)}\\ = & \ M\cdot \left(M\cdot H^{(0)}\cdot W^{(1)}\right)\cdot W^{(2)}\\ = & \ M^{2}\cdot H^{(0)}\cdot W^{(1)}\cdot W^{(2)}, \end{aligned}\end{align*}

where $H^{(2)}\in \mathbb{R}^{(N_{m}+N_{d})\times d}$ and $W^{(2)}\in \mathbb{R}^{d\times d}$.

Similarly, the $l$-layer convolution is denoted as follows: 


(18) \begin{align*}& \begin{aligned} H^{(l)} = & \ M\cdot H^{(l-1)}\cdot W^{(l)}\\ = & \ M\cdot \left(M\cdot H^{(l-2)}\cdot W^{(l-1)}\right)\cdot W^{(l)}\\ = & \ M^{l}\cdot H^{(0)}\cdot W^{(1)}\cdots W^{(l)}. \end{aligned}\end{align*}

Finally, MGCNSS could establish $l$ feature matrixes, corresponding to the meta-paths with $l$ different lengths. Notably, lower-order convolutional layers tend to focus on the neighbors of miRNAs or diseases, while higher-order convolutional layers could capture the relationships from long distances. Then, we apply attention coefficients here to combine the $l$ feature matrices with different weights: 


(19) \begin{align*}& H=\gamma_{1}\cdot H^{(1)} + \gamma_{2}\cdot H^{(2)} + \cdots + \gamma_{l}\cdot H^{(l)},\end{align*}

where $H$ is the final output of the multi-layer graph convolution module and the ultimate feature matrix of miRNAs and diseases.

**Figure 2:**
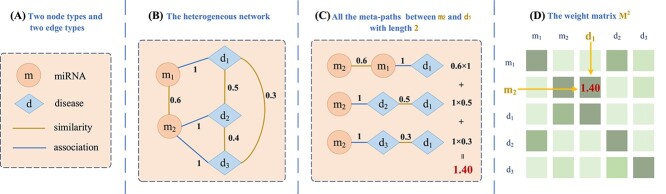
A toy example for learning the importance of meta-paths. (**A**) There are two node types and two edge types. (**B**) The miRNA–disease heterogeneous network, where the numbers on yellow edges and blue edges represent the similarities between the different nodes and the association relationships, respectively. (**C**) For the meta-paths with length equal to 2, we display all the paths from node $m_{2}$ to $d_{1}$. For each path, we first multiply the weights of its two edges to measure the total weight of the corresponding path. Then, MGCNSS could construct the weight matrix $M_{c}^{2}$ in terms of the meta-paths with length 2. (**D**) The integrated result from $m_{2}$ to $d_{1}$ is shown in the weight matrix $M_{c}^{2}$.

Figure 2 is a toy example demonstrating the process of the multi-layer graph convolution in capturing the semantics of meta-paths with length 2 automatically.

#### Negative sample selection strategy

In the association prediction tasks, the quality of negative samples always affects the performance of the prediction models [48]. Positive samples can be directly collected, but obtaining the ground-truth negative samples is a challenging task [49]. Therefore, most current approaches always treat the known miRNA–disease associations as the positive samples, while the remained relationships are regarded as the unlabeled samples. Moreover, previous research usually randomly selects samples with a certain number from unlabeled samples to form the negative sample set [50]. Generally, this negative selection strategy may introduce some dirty samples, which may interfere with the model training process and reduce the prediction accuracy.

To solve this problem, we propose a distance-based negative sample selection strategy including cosine distance and Euclidean distance to select reliable negative samples. Firstly, we combine the k-means clustering algorithm [51] to generate a centroid vector $C_{p}$ for the positive sample set $P_{set}$ and a centroid vector $C_{u}$ for the unlabeled sample set $U_{set}$. Specifically, $C_{p}$ and $C_{u}$ are calculated by the following formula: 


(20) \begin{align*} & C_{p}=1/| P_{set}\vert \ast\sum_{i=0}^{\mid P_{set} \mid }F_{v_{i}} \end{align*}

(21) \begin{align*} & C_{u}=1/| U_{set}\vert \ast\sum_{j=0}^{| U_{set}\vert }F_{v_{j}},\end{align*}

where $v_{i}$ denotes the $i$th miRNA–disease pair and $F_{v_{i}}$ denotes its vector representation. Specifically, $F_{v_{i}}\in \mathbb{R}^{1\times{(N_{m}+N_{d})}}$ is formed by concatenating the feature vectors of miRNA–disease pairs from the positive sample set. Similarly, $F_{v_{j}}\in \mathbb{R}^{1\times (N_{m}+N_{d})}$ is the concatenated vectors in unlabeled sample set.

Next, we compare the cosine similarity (CS) between each unlabeled sample and these two centroid vectors $C_{p}$ and $C_{u}$. For one sample $v_{i} \in U_{set}$, if $CS_{p}$ is greater, we put it into a potential positive sample set $P_{l}$. Conversely, if $CS_{u}$ is greater, it will be put into a potential negative sample set $N_{l}$. The formulas for CS calculation for $CS_{p}$ and $CS_{u}$ are defined as follows: 


(22) \begin{align*} & CS_{p}=cosine\left(F_{v_{i}},C_{p}\right), \end{align*}

(23) \begin{align*} & CS_{u}=cosine\left(F_{v_{i}},C_{u}\right).\end{align*}

In this manner, we can obtain $P_{l}$ set and $N_{l}$ set and calculate their corresponding novel centroid vectors $C_{p}^{{\prime}}$ and $C_{u}^{{\prime}}$ with Equations (21) and (22), respectively. Then we compare the samples $v_{i} \in U_{set}$ with the new centroid vectors $C_{p}^{{\prime}}$ and $C_{u}^{{\prime}}$ by Euclidean similarity (ES) measurement and divide them accordingly. The formula for calculating the ES is as follows: 


(24) \begin{align*} & ES_{p}=1\big/\left(1 + \| F_{v_{i}}-C_{p}^{{\prime}}\Vert^{2}\right), \end{align*}

(25) \begin{align*} & ES_{u}=1\big/\left(1 + \| F_{v_{i}}-C_{u}^{{\prime}}\Vert^{2}\right),\end{align*}

MGCNSS divides the samples in $U_{set}$ into the $P_{l}^{{\prime}}$ and $N_{l}^{{\prime}}$ according to their $ES_{p}$ and $ES_{u}$ values and starts the next iteration.

**Figure 3:**
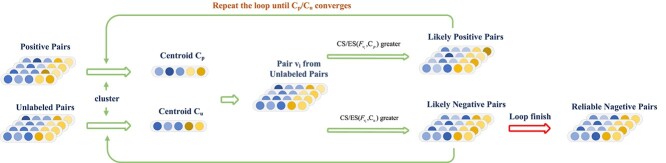
The process of negative sample selection strategy. First, MGCNSS generates centroid vectors $C_{p}$ and $C_{u}$ from positive samples and the remaining unlabeled samples, respectively. Then, MGCNSS calculates the CS between each sample in the unlabeled sample set and the centroid vectors $C_{p}$ and $C_{u}$, respectively. Based on the CS, we could divide the unlabeled samples into two groups, Likely Positive Pairs (LP) and Likely Negative Pairs (LN). Next, we update the two centroid vectors using LN and LP. Moreover, MGCNSS adopts ES to repeat these steps until the centroid vectors $C_{p}$ and $C_{u}$ converge. Finally, we regard LN as the reliable negative sample set.

This process is repeated until the centroids converge, which meets the following conditions: 


(26) \begin{align*} & \| C_{p} -C_{p}^{{\prime}}\Vert_{F} \leq 10^{-3} \end{align*}

(27) \begin{align*} & \|C_{u} -C_{u}^{{\prime}} \Vert_{F} \leq 10^{-3}\end{align*}

where $\left \lVert \cdot \right \rVert $ is the $Frobenius$ norm operation. The $N_{l}^{{\prime}}$ in the last iteration is treated as the final reliable negative sample set denoted as $N_{l}$. The selection process is shown in Figure 3 and the algorithm is presented in Algorithm 1. 


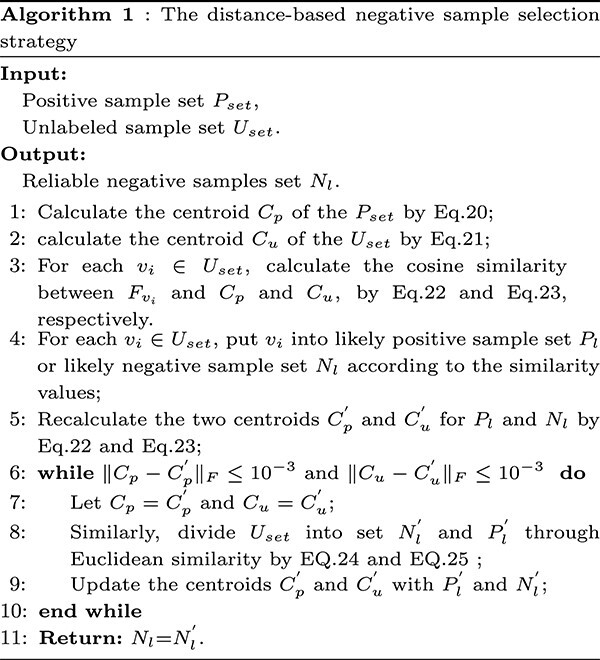


### Model training

Finally, we train MGCNSS by minimizing the binary cross-entropy loss function to optimize the model parameters, which is formulated as follows: 


(28) \begin{align*}& \mathcal{L}=-\!\!\sum_{(m,d)\in P}\!{log\ \sigma \left(\langle H_{m},H_{d}\rangle \right)}\enspace -\!\!\!\!\!\sum_{(m^{{\prime}}\!,d^{{\prime}})\in N_{l}}\!\!\!\!\!\!{log\ (1- \sigma \left(\langle H_{m^{{\prime}}},H_{d^{{\prime}}}\rangle \right))},\end{align*}

where $H_{m}$ represents the $m$th row vector of the feature matrix obtained from the convolutional layer, $\sigma $ denotes the sigmoid function, and $\langle H_{m}, H_{d}\rangle $ represents the inner product of $H_{m}$ and $H_{d}$.

### Time complexity analysis

Here we analyze the time complexity of MGCNSS. As is shown in Figure 1, MGCNSS mainly has three modules, which are multi-source data fusion, multi-layer graph convolution and negative sample selection. Suppose that there are $m$ miRNAs and $n$ diseases, MGCNSS has to measure the similarities between all the miRNAs and diseases, and the time complexity is $O(m^{2}+n^{2})$. The time complexity for integrating different miRNA and disease similarity networks is $O(3m^{2}+3n^{2})$. The time complexity for multi-layer graph convolution is $O(mn*mn*d)$, where $d$ is the dimension of embeddings of miRNAs and diseases. The time complexity for negative sample selection is $O(k*(m*n)/2)$, where $k$ is the number of iterations. As a result, the total complexity for MGCNSS is $O(m^{2}+n^{2})$+$O(mn*mn*d)$+ $O(k*(m*n)/2)$, which is equivalent $O(mn*mn*d)$. Since $d$ is a constant, the final time complexity for MGCNSS is $O(m^{2}n^{2})$. In this study, the running time for MGCNSS is about 5 s to complete a five-fold crossover experiment and the running time for performing 2000 epochs is about 146 minutes.

## RESULTS

In this section, we first briefly introduce the implementation details and evaluation metrics used in this study. Then the comparison results of MGCNSS as well as the baselines are well presented. After that, the ablation experiments and the parameter sensitivity experiments are demonstrated. Last, the case studies are displayed.

### Implementation details and evaluation metrics

For MGCNSS, the embedding size of the miRNAs and diseases for prediction is 256, the learning rate is 0.0005 and the weight decay is 0.0005. Besides, the number of training epochs is uniformly 2000. In the multi-layer graph convolution module, the number of convolution layers $l$ is 2.

Meanwhile, to comprehensively evaluate the performance of the prediction models, we establish two types of experiment datasets according to the ratio between the number of positive and negative pairs. Specifically, on the balanced dataset, the ratio between the number of positive and negative samples is 1:1. On the imbalanced dataset, the ratio between the number of positive and negative samples is 1:5 and 1:10.

Finally, we employ the widely used evaluation metrics widely used [52] to evaluate the performance of MGCNSS and the comparison approaches, which are accuracy (ACC), area under receiver operating characteristic curves (AUC) and area under the precision-recall curves (AUPR). Specifically, AUC is commonly employed to evaluate the stability of the prediction model, while ACC is adopted to measure the ability of the prediction model in the accuracy predicting aspect. AUPR is another crucial metric for evaluating the performance of prediction models on the imbalanced dataset.

**Figure 4:**
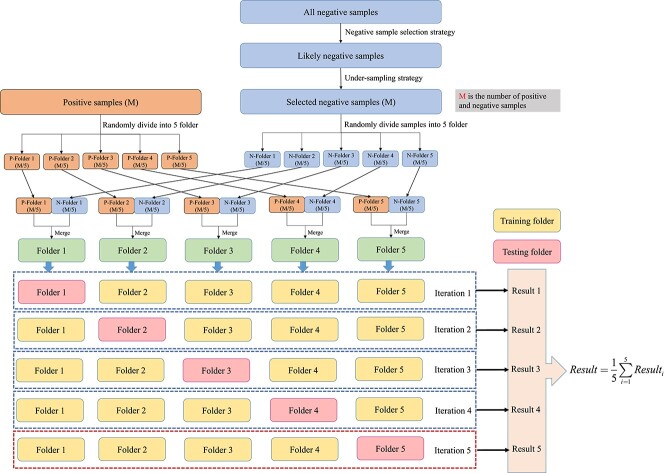
The 5-folder cross-validation strategy used in this study.

Besides, we employ the 5-folder cross-validation strategy (5-CV) to further evaluate the performance of MGCNSS. Figure 4 demonstrates the process of 5-CV on the balanced dataset. MGCNSS first employs the negative sample selection strategy to choose the likely negative samples from all the negative samples. Then MGCNSS selects $M$ samples from the likely negative sample set. The number of positive samples is also $M$. After that, MGCNSS divides the positive and selected negative samples into five folders randomly and performs the 5-CV experiment. Each testing folder will be used to evaluate the performance of MGCNSS in turn for each iteration. Finally, MGCNSS adopts the average value of each iteration as the evaluation result for the corresponding epochs. The description above is the execution process for the one-time epoch. With the increase of the epoch number, BCE loss will be converged and MGCNSS could get the best performance and the corresponding hyperparameters.

Moreover, the selection for the hyperparameters used in this study is based on the training dataset. The negative sample-selection strategy is also applied to the training dataset as well as the test dataset. Besides, in the parameter sensitivity analysis section, the presented results are derived from the test dataset.

### Comparison with other baseline models

Here, we mainly select eleven competitive methods to compare with the proposed model, which are SVM [18], RF [53], XGBoost [54], GCN [55], GAT [56], DTIGAT [57], DTICNN [58], NeoDTI [59], MSGCL [3], AMHMDA [35] and GATMDA [13]:

- SVM [18]: is a classic supervised learning algorithm, and we feed the learned embeddings of drugs and targets directly for MDA prediction.
- RF [53]: is an ensemble learning method that combines multiple decision trees for DTI prediction.
- XGBoost [54]: is a widely used gradient boosting framework in which the features of miRNAs and diseases are fed directly for MDA prediction.
- GCN [55]: is a neural network architecture designed for graph-structured data, which is employed to learn the embeddings of miRNAs and diseases for MDA association prediction.
- GAT [56]: is also a neural network architecture that utilizes the attention mechanism in the feature learning process.
- DTIGAT [57]: is an end-to-end framework that assigns different weights to node neighbors with the self-attention mechanisms for DTI prediction.
- DTICNN [58]: adopts RWR algorithm to extract features and employs a denoising auto-encoder for dimensionality reduction for MDA predictions.
- NeoDTI [59]: is an end-to-end model that could integrate different information and automatically learn topology-preserve representations.
- MSGCL [3]: adopts the multi-view self-supervised contrastive learning for MDA prediction that could enhance the latent representation by maximizing the consistency between different views.
- AMHMDA [35]: applies the attention-aware multi-view similarity strategy to learn the embeddings of nodes from the heterogeneous hyper-graph to predict the miRNA–disease associations.
- GATMDA [13]: could both fuse linear and non-linear embeddings of miRNAs and diseases and adopt the RF model to complete the prediction task.

We first perform the comparison experiment on the balanced dataset, in which the ratio between the number of positive and negative samples is 1:1. The results are shown in Table 1. It can be observed that MGCNSS outperforms all baseline methods significantly in this scenario. Specifically, the results of MGCNSS on AUC, ACC and AUPR metrics are 0.9874, 0.9453 and 0.9882 respectively. Besides, XGBoost wins the second rank on AUC and AUPR, respectively, and the corresponding values are 0.9353 and 0.9355. Meanwhile, GAT gets the second highest score on ACC metric and its value is 0.8647.

**Table 1:** The evaluation results of MGCNSS and baseline methods with 1:1 ratios on AUC, ACC and AUPR metrics.

Model	AUC	ACC	AUPR
SVM [18]	0.8990${\pm }$0.0012	0.8051${\pm }$0.0132	0.9059${\pm }$0.0099
RF[53]	0.9255${\pm }$0.0021	0.8412${\pm }$0.0023	0.9201${\pm }$0.0027
XGBoost [54]	0.9353${\pm }$0.0121	0.8637${\pm }$0.0142	0.9355${\pm }$0.0019
GCN [55]	0.8340${\pm }$0.0012	0.7840${\pm }$0.0024	0.8715${\pm }$0.0042
GAT [56]	0.8734${\pm }$0.0023	0.8012${\pm }$0.0032	0.8703${\pm }$0.0041
DTIGAT [57]	0.9153${\pm }$0.0004	0.8647${\pm }$0.0054	0.9109${\pm }$0.0032
DTICNN [58]	0.9057${\pm }$0.0032	0.8230${\pm }$0.0043	0.8979${\pm }$0.0042
NeoDTI [59]	0.8301${\pm }$0.0153	0.7915${\pm }$0.0041	0.8568${\pm }$0.0021
MSGCL [3]	0.8370${\pm }$0.0042	0.7763${\pm }$0.0012	0.9162${\pm }$0.0031
MHMDA [35]	0.9176${\pm }$0.0054	0.8063${\pm }$0.0165	0.9167${\pm }$0.0147
GATMDA [13]	0.9279${\pm }$0.0120	0.8471${\pm }$0.0231	0.9269${\pm }$0.0039
MGCNSS(ours)	**0.9874${\pm }$0.0078**	**0.9453${\pm }$0.0089**	**0.9882${\pm }$0.0013**
MGCNSS w/o NSST	0.94371${\pm }$0.0057	0.8859${\pm }$0.0112	0.9125${\pm }$0.0188

Note: MGCNSS w/o NSST is the variant of MGCNSS and we only compare it with MGCNSS. The best results are marked in bold and the second best is underlined.

Moreover, we vary the ratio between the number of positive and negative samples, which are 1:5 and 1:10. The corresponding results are also listed in Table 2. From the results, we can see that the proposed method wins the best performance on all the evaluation metrics. Specifically, the results of MGCNSS with the 1:5 ratio are 0.9861, 0.9586 and 0.9758 on AUC, ACC and AUPR metrics, while those of MGCNSS with the 1:10 ratio are 0.9871, 0.9786 and 0.9385 on the corresponding metrics. Besides, XGBoost gets the second best on AUC and AUPR with the 1:5 ratio and 1:10 ratio, on ACC with the 1:10 ratio. Meanwhile, DTIGAT ranks second on ACC with a 1:5 ratio.

**Table 2:** The evaluation results of MGCNSS and baseline methods on 1:5 and 1:10 ratios

Model	AUC		ACC		AUPR	
	1:5	1:10	1:5	1:10	1:5	1:10
SVM [18]	0.9105${\pm }$0.0012	0.9010${\pm }$0.0019	0.8963${\pm }$0.0031	0.9129${\pm }$0.0025	0.7440${\pm }$0.0018	0.6093${\pm }$0.0076
RF [53]	0.9279${\pm }$0.0041	0.9291${\pm }$0.0067	0.9078${\pm }$0.0121	0.9402${\pm }$0.0067	0.7721${\pm }$0.0032	0.6861${\pm }$0.0038
XGBoost [54]	0.9404${\pm }$0.0042	0.9579${\pm }$0.0022	0.9179${\pm }$0.0087	0.9566${\pm }$0.0045	0.8511${\pm }$0.0031	0.8099${\pm }$0.0041
GCN [55]	0.8291${\pm }$0.0098	0.8410${\pm }$0.0033	0.9122${\pm }$0.0044	0.9435${\pm }$0.0023	0.6367${\pm }$0.0089	0.5115${\pm }$0.0066
GAT [56]	0.8637${\pm }$0.0011	0.8682${\pm }$0.0042	0.8509${\pm }$0.0043	0.9040${\pm }$0.0024	0.6841${\pm }$0.0032	0.4669${\pm }$0.0078
DTIGAT [57]	0.9103${\pm }$0.0043	0.9085${\pm }$0.0113	0.9198${\pm }$0.0098	0.9381${\pm }$0.0120	0.8009${\pm }$0.0321	0.6987${\pm }$0.0132
DTICNN [58]	0.9066${\pm }$0.0042	0.9087${\pm }$0.0313	0.8903${\pm }$0.0023	0.9281${\pm }$0.0038	0.7119${\pm }$0.0138	0.5915${\pm }$0.0201
NeoDTI [59]	0.8430${\pm }$0.0029	0.8287${\pm }$0.0031	0.8144${\pm }$0.0065	0.8597${\pm }$0.0087	0.6531${\pm }$0.0043	0.5219${\pm }$0.0098
MSGCL [3]	0.8371${\pm }$0.0077	0.8385${\pm }$0.0098	0.8042${\pm }$0.0099	0.8584${\pm }$0.0043	0.7391${\pm }$0.0187	0.6271${\pm }$0.0032
AMHMDA [35]	0.9178${\pm }$0.0163	0.9144${\pm }$0.0067	0.8927${\pm }$0.0181	0.9320${\pm }$0.0039	0.7431${\pm }$0.0229	0.6395${\pm }$0.0312
GATMDA [13]	0.9247${\pm }$0.0077	0.9274${\pm }$0.0392	0.9170${\pm }$0.0124	0.9465${\pm }$0.0191	0.8048${\pm }$0.0521	0.7253${\pm }$0.0391
MGCNSS(ours)	**0.9861${\pm }$0.0016**	**0.9871${\pm }$0.0025**	**0.9586${\pm }$0.0178**	**0.9786${\pm }$0.0092**	**0.9758${\pm }$0.0077**	**0.9385${\pm }$0.0112**
MGCNSS w/o NSST	0.9439${\pm }$0.0066	0.9442${\pm }$0.0188	0.9082${\pm }$0.0076	0.9311${\pm }$0.0102	0.8615${\pm }$0.0542	0.8053${\pm }$0.0201

Note: Since MGCNSS w/o NSST is the variant of MGCNSS, we only compare it with MGCNSS. The best results are marked in bold and the second best is underlined.

Meanwhile, to fully investigate the predictive performance of MGCNSS when the negative sample selective strategy does not include information from the test set and only includes information from the training set, we perform this ablation experiment named MGCNSS w/o NSST (**MGCNSSwithoutN**egative **S**ample **S**elective strategy on **T**esting set). Specifically, the negative sample selective strategy only applies to the training sample set in the training process and does not apply to the testing sample set in the testing process. We perform the corresponding experiments under different ratios (1:1, 1:5 and 1:10) and their corresponding results are displayed in Tables 1 and 2.

The results demonstrate that the performance of MGCNSS w/o NSST is inferior to MGCNSS. For example, in Table 1, the values of MGCNSS w/o NSST on AUC, ACC and AUPR are 0.9437, 0.8859 and 0.9125, which are lower than those of MGCNSS by 3.1, 6.3 and 7.6% respectively. The metric values of MGCNSS w/o NSST in Table 2 are also inferior to those of MGCNSS and here we don’t repeat these results anymore. In conclusion, the results presented in Tables 1 and 2 could illustrate that the negative sample selection strategy is essential for MGCNSS. The results of this ablation experiment demonstrate that the proposed negative sample selection strategy affects the performance of MGCNSS.

### The ablation experiments

#### Multi-source data fusion, multi-layer graph convolution and negative sample selection

In MGCNSS, there are three essential modules which are the multiple similarities integration module (denoted as MI, see Figure 1A), the meta-path-based multi-layer graph convolution module (denoted as MP; see Figure 1B) and the negative sample selection module (denoted as SS; see Figure 1C). Here, we conduct ablation experiments to investigate the effectiveness of these modules. Specifically, the ablation experiments are performed with MGCNSS without MI (MGCNSS w/o MI), MGCNSS without MP(MGCNSS w/o MP), MGCNSS without SS (MGCNSS w/o SS) and MGCNSS.

**Figure 5:**
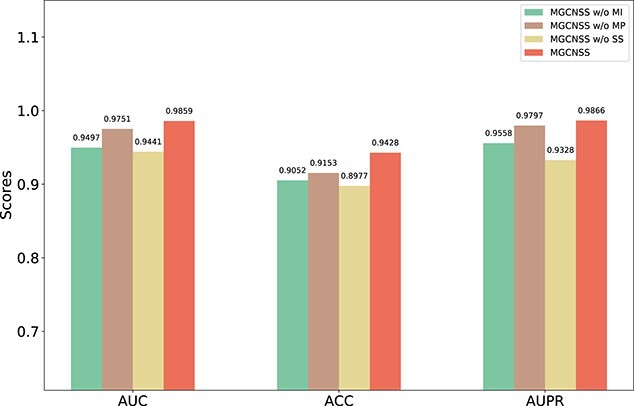
The ablation experimental results of MGCNSS w/o MI, MGCNSS w/o MP, MGCNSS w/o SS and MGCNSS with 1:1 ratio.

The result shows that both MI, MP and SS are essential for MGCNSS. Specifically, MGCNSS outperforms MGCNSS w/o MI, MGCNSS w/o MP and MGCNSS w/o SS by 3.81, 1.11 and 4.42% on the AUC metric, respectively. For the ACC metric, MGCNSS outperforms MGCNSS w/o MI, MGCNSS w/o MP and MGCNSS w/o SS by 4.15, 3.00 and 5.02%. Besides, MGCNSS outperforms MGCNSS w/o MI, MGCNSS w/o MP and MGCNSS w/o SS by 3.22, 0.70 and 5.76% on the AUPR metric, respectively. MGCNSS achieves the best performance on all three metrics. The corresponding results are shown in Figure 5.

**Figure 6:**
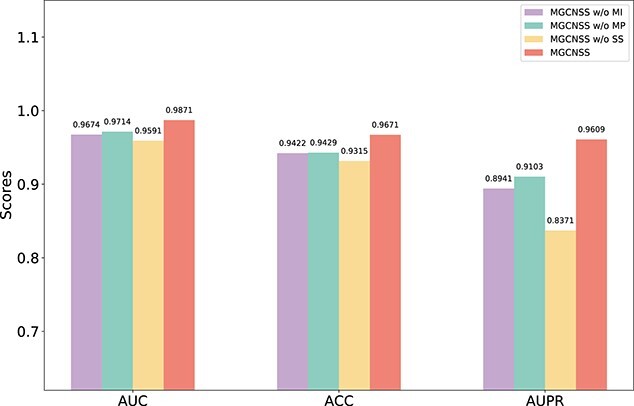
The ablation experimental results of MGCNSS w/o MI, MGCNSS w/o MP, MGCNSS w/o SS and MGCNSS with 1:5 ratio.

Besides, we conducted validation on the imbalanced dataset. Specifically, the experiments were performed with the 1:5 ratio and the results are shown in Figure 6. The results demonstrate that on the imbalanced dataset, the SS module significantly improves the model performance. The values of MGCNSS on AUC, ACC and AUPR metrics are 0.9871, 0.9671 and 0.9609, respectively. Compared with the variants of MGCNSS, the performance of MGCNSS is competitive. The results shown in Figure 6 further show SS, MP and MI modules are all essential in improving the prediction accuracy. Meanwhile, the results demonstrate that meta-path-based multi-layer graph convolution plays an essential role in improving the performance of MGCNSS.

#### Different similarities of miRNAs and diseases in multi-source data fusion

**Figure 7:**
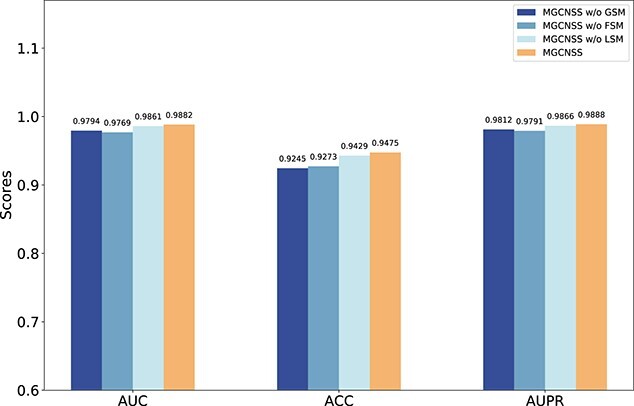
The ablation experimental results of MGCNSS w/o FSM, MGCNSS w/o GSM, MGCNSS w/o LSM and MGCNSS.

In the multi-source data fusion module (Figure 1A), MGCNSS integrates three different types of similarities, which are miRNA functional similarity and disease semantic similarity (denoted as FSM), GIP Kernel similarity of miRNAs and diseases (denoted as GSM), lncRNA-based similarity of miRNAs and diseases (denoted as LSM), respectively. This ablation experiment is formulated as MGCNSS w/o FSM, MGCNSS w/o GSM, MGCNSS w/o LSM and MGCNSS, and their corresponding results on the balanced dataset are shown in Figure 7.

The results shown in Figure 7 indicate that MGCNSS wins the highest scores on AUC, ACC and AUPR metrics. To be specific, MGCNSS gets the highest scores on the AUC, ACC and AUPR metrics and their corresponding scores are 0.9882, 0.9475 and 0.9888. Compared with MGCNSS, the scores of MGCNSS w/o GSM, MGCNSS w/o FSM and MGCNSS w/o LSM on ACC are 2.4, 2.1 and 0.4% lower than MGCNSS. The results illustrate that these three types of similarities are all essential for MGCNSS.

### The performance of MGCNSS based on meta-paths with different lengths

To fully evaluate the effect of meta-paths with different lengths on MGCNSS, we divide the meta-paths into different combinations {1}, {2}, {3}, {1,2,3} and {1,2}, which are shown in Table 3. Specifically, the corresponding results for each combination are also presented in Table 3.

**Table 3:** The performance of MGCNSS based on meta-paths with different lengths

Meta-path length	AUC	ACC	AUPR
{1}	0.9819	0.9355	0.9835
{2}	0.9160	0.8573	0.9192
{3}	0.9157	0.8600	0.9340
{1,2,3}	0.9643	0.9088	0.9722
{1,2}	0.9874	0.9453	0.9882

MGCNSS on meta-path with lengths {1,2} wins the best performance, and the AUC, ACC and AUPR values are 0.9874, 0.9453 and 0.9882 respectively. Besides, MGCNSS with length {1} achieves the second rank on AUC, ACC and AUPR metrics are 0.9819, 0.9355 and 0.9835. Meanwhile, we also find that the performance of MGCNSS on meta-paths with length {1,2,3} is competitive. This may be because embeddings learned from the meta-path with length 3 may have noise, which leads to a decrease in the performance of MGCNSS. It is worth noting that meta-paths with length {1,2} have the greatest impact on the performance of MGCNSS.

### Parameter sensitivity analysis

In this section, we first conduct the parameter sensitivity analysis experiments, which are the learning rates, embedding sizes, and the number of graph convolution layers. Then, we introduce the procedure for selecting hyperparameters in Equations (12) and (13).

**Figure 8:**
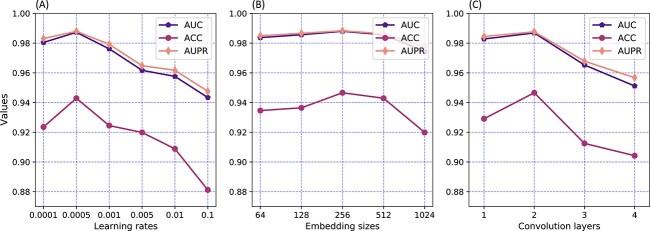
The parameter sensitivity analysis with different learning rates, embedding sizes and the number of convolution layer.

The first parameter is the learning rate, which is a hyperparameter that controls how much to change one model in response to the estimated error [60]. It is one crucial task to choose a proper learning rate for MGCNSS. In this study, we choose the learning rate from 0.0001, 0.0005, 0.001, 0.01 and 0.1, respectively, and their corresponding results are shown in Figure 8(A). MGCNSS achieves the best performance on all metrics when the learning rate is 0.0005. The results show that the performance of MGCNSS gets better when the learning rate increases from 0.0001 to 0.0005, while the metrics decrease when the learning rate ranges from 0.0005 and 0.1. As a result, MGCNSS adopts 0.0005 as its best learning rate.

The second parameter is the embedding size of miRNAs and diseases, which is essential for the performance of MGCNSS. In this study, we vary the embedding size from 64, 128, 256, 512 and 1,024 and the corresponding results are shown in Figure 8(B). It can be seen that MGCNSS gets its best performance when the embedding size is 256. In conclusion, we adopt 256 as the best embedding size for MGCNSS.

The third parameter is the number of convolution layers, which affects the performance of MGCNSS. Here we choose the number of graph convolution layers from 1, 2, 3 and 4 and then obtain the AUC, ACC and AUPR values. Results shown in Figure 8(C) illustrate that the proposed model wins the highest scores when the number of graph convolution layers is 2. Notably, the performance of MGCNSS begins to decline when the number of convolutional layers is larger than 2. It may be that 1-length and 2-length meta-paths could have been able to fully capture the semantics between nodes, while the longer paths would not be helpful for the embedding learning of miRNAs and diseases. Therefore, the number of graph convolutional layers is set to 2.

**Figure 9:**
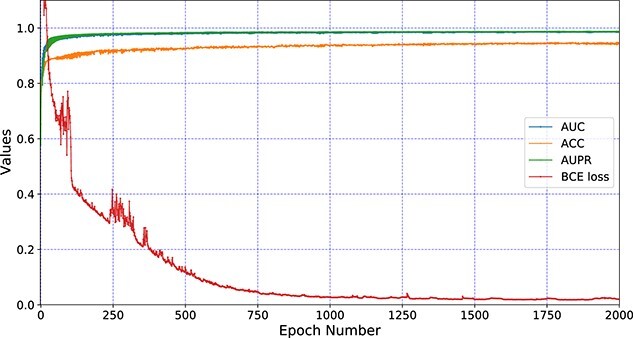
The change of AUC, ACC and AUPR values accompanied by the BCE loss under different epochs.

Besides, for hyperparameters in Eq. 12 and Eq 13, MGCNSS adopts the BCEloss to select their proper values. As is shown in Figure 9, we can see that the BCE loss tends to converge with the increase of epoch number. Specifically, when the epoch number is larger than 1500, the BCE loss is almost converged and the corresponding AUC, ACC and AUPR values change in a small range.

**Figure 10:**
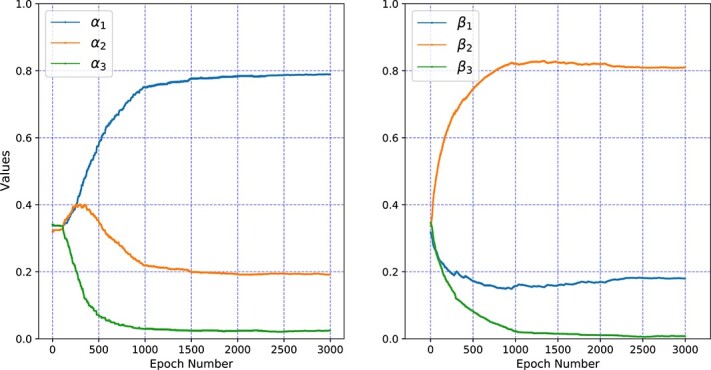
The change of different hyperparameters under different epochs.

Meanwhile, Figure 10 presents the correspondence values of hyperparameters in Equations (12) and (13) with the increase of the epoch number. In this study, MGCNSS chooses the $\alpha _{1}$, $\alpha _{2} $, $\alpha _{3}$ and $\beta _{1}$$\beta _{2}$, and $\beta _{3} $ value when the epoch number is equal to 2000. Their corresponding values are 0.77, 0.19, 0.04 and 0.16, 0.82, 0.02, which indicates that the miRNA functional similarity network ($\alpha _{1} $) and Gaussian kernel-based disease similarity network ($\beta _{2}$) have relatively higher weights.

### The performance of MGCNSS under different negative sample selection strategy

**Figure 11:**
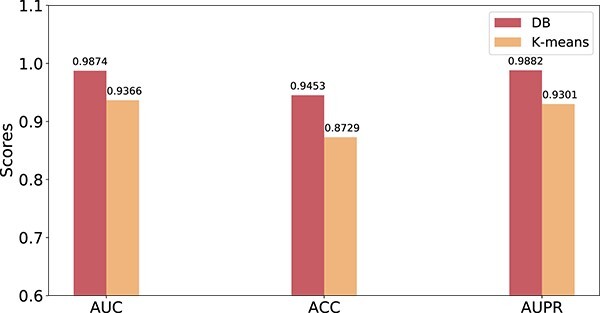
The results of MGCNSS under different negative sample selection strategies.

As we know, the negative sample selection strategy is crucial to the performance of MGCNSS. Hence, there are some other negative sample selection strategies [5, 60]. Here, we choose the k-means clustering strategy in [5] and compare it with our distance-based (DB) selection strategy. The corresponding results are displayed in Figure 11.

This study employs the AUC, ACC and AUPR metrics to evaluate the performance of MGCNSS with different negative sample selection strategies. We can see that MGCNSS with our DB strategy achieves higher scores, and the AUC, ACC and AUPR values are 0.9874, 0.9453 and 0.9882, respectively, while MGCNSS with the k-means strategy gets 0.9366, 0.8729 and 0.9301 on AUC, ACC and AUPR, respectively. The results in this sub-experiment further illustrate the effectiveness of the proposed negative sample selection strategy.

### The results of MGCNSS and other approaches under statistical significance test

The statistical significance test is another commonly used manner to verify the resulting stability of each prediction model. Here, we employ the paired *t*-test model [61] to perform the significance analysis. Specifically, the results of MGCNSS and the baselines are paired in terms of AUC, ACC and AUPR metric values respectively. In the paired *t*-test, we set the significance level as 0.05. The null hypothesis ($H_{0}$) is that the performance of MGCNSS is not significantly better than baseline models on the given evaluation metrics, while the hypothesis ($H_{1}$) is that MGCNSS is significantly better than baseline models. If the *P*-value is less than 0.05, we will reject $H_{0}$ and accept $H_{1}$. The corresponding results have been fully displayed in Table 4, which indicates that the performance of MGCNSS is superior to all the baseline approaches.

**Table 4:** The statistical significance analysis for MGCNSS and baseline approaches

Model comparison	AUC	ACC	AUPR
MGCNSS vs SVM [18]	3.3E-10	8.2E-09	2.8E-10
MGCNSS vs RF [53]	1.7E-09	2.4E-08	7.2E-10
MGCNSS vs XGBoost [54]	4.9E-09	5.7E-08	2.1E-09
MGCNSS vs GCN [55]	5.7E-11	4.5E-09	8.3E-11
MGCNSS vs GAT [56]	2.1E-10	6.7E-09	9.0E-11
MGCNSS vs DTIGAT [57]	1.0E-09	6.0E-08	4.8E-10
MGCNSS vs DTICNN [58]	5.7E-10	1.3E-08	2.4E-10
MGCNSS vs NeoDTI [59]	5.3E-11	5.0E-09	5.2E-11
MGCNSS vs MSGCL [3]	6.2E-11	3.6E-09	5.7E-10
MGCNSS vs AMHMDA [35]	1.3E-09	7.8E-09	6.0E-10
MGCNSS vs GATMDA [13]	1.3E-09	3.1E-08	1.1E-09

### Visualization for the embeddings of miRNA–disease pairs learned by MGCNSS

**Figure 12:**
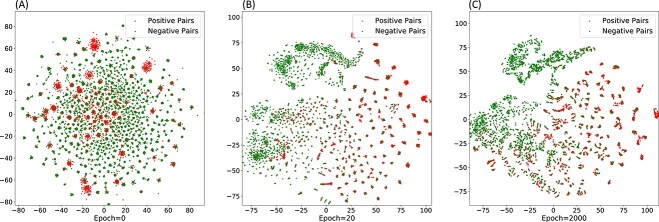
Visualization for the embeddings of miRNA–disease pairs learned by MGCNSS under different epochs.

To better demonstrate the effectiveness of MGCNSS, we display the embedding learning process of miRNA–disease pairs. Similar to the SCSMDA method [60], the positive pairs (red points) and negative pairs (blue points) are pre-selected and their embeddings are visualized by t-SNE tool [62] under different epochs. The results are shown in Figure 12.

It can be seen that the boundary between positive and negative pairs seems in *chaos* when the epoch is 0. With the increase of epoch number, the embeddings of positive and negative pairs become clear gradually. When the epoch number reaches 2000, the positive and negative pairs are almost separated with one distinct boundary. Meanwhile, It is worth noting that even if the epoch number is 2000, there may still be overlapping between positive and negative pairs, indicating the presence of significant challenges in the prediction task of miRNA–disease associations. Overall, the results confirm the powerful ability of MGCNSS to learn the discriminative embeddings of miRNAs and diseases.

**Figure 13:**
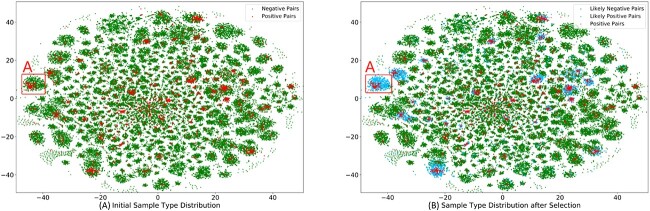
Comparison results of MGCNSS without and with negative sample screening strategy.

Besides, to fully demonstrate the positions of positive samples and negative samples after executing our negative sample selection strategy, we visualize their 2D projections in Figure 13. Specifically, without the negative sample selection strategy, MGCNSS will divide the training samples into two categories, which are the positive samples (red points) and negative samples (blue points) in Figure 13(A). Meanwhile, after executing the proposed distanced-based negative sample selection strategy, MGCNSS divides the training samples into three categories (see Figure 13B), which are positive samples (red points), likely negative samples (green points) and likely positive samples (blue points).

Specifically, the local area A in Figure 13(A) contains the positive pairs and negative pairs. If there is no negative sample selection strategy, all the negative samples in area A could be the candidate negative samples. In fact, since the local area A gathers many ground-truth positive samples, the negative samples having a close distance to the ground-truth positive samples may not be the ground-truth negative samples. In other words, they may be the likely positive samples and should not be treated as candidate negative samples for training and testing. Correspondingly, in Figure 13(B), because of the negative sample selection strategy, MGCNSS marked the negative samples in local area A as the likely positive samples (blue points), not the negative samples for training and testing. In this way, MGCNSS will select the negative samples from the likely negative samples instead of the likely positive samples. As a result, MGCNSS will establish a high-quality negative sample set, which will improve its performance. The extensive results in the former section could further verify the effectiveness of the negative sample selection strategy.

### Case study

We conduct the case study to further evaluate the performance of MGCNSS, which is similar to our previous research [29]. Firstly, we train MGCNSS with all the miRNA–disease associations in the HMDDv2.0 dataset. Then, we predict the potential associated miRNAs for the selected diseases. After that we screen out the top 50 predicted miRNAs based on their corresponding scores. To validate these predicted associations, we search the evidence based on HMDDv4.0 [63] and dbDEMC [64] database. Currently, in HMDDv4.0, there are 53 553 miRNA–disease association entries which include 1817 human miRNA genes, 79 virus-derived miRNAs and 2379 diseases from 37 022 papers. Besides, dbDEMC is designed to store and display differentially expressed miRNAs in cancers detected by high- and low-throughput strategies. Its current version contains 2584 miRNAs and 40 cancer types for humans.

Specifically, disease colon neoplasms and esophageal neoplasms are selected for validation. Colon neoplasms is a common malignant tumor that occurs in the colon of the digestive tract [65]. It ranks third in the incidence of gastrointestinal tumors and is increasing year by year, causing more than one million cases and 500 000 deaths annually. We first investigate the miRNAs associated with colon neoplasms, and the results are shown in Table 5. The results demonstrate that the top 50 predicted miRNAs have all been confirmed by HMDD or dbDEMC. As another high-incidence disease, esophageal neoplasm is a malignant tumor occurring in the esophageal tissue. Its morbidity and mortality are high, ranking 8th and 6th in all types of cancer, respectively [66]. Early diagnosis is beneficial to improving the survival rate of patients. The corresponding results (Table 6) show that 49 of the top 50 can be verified by HMDD or dbDEMC. The results of the case study fully illustrate the ability of MGCNSS to detect novel associations between miRNAs and diseases.

**Table 5:** Top 50 colon neoplasms-related miRNAs predicted by MGCNSS

RANK	miRNA name	Evidence	RANK	miRNA name	Evidence
1	hsa-mir-21	confirmed	26	hsa-mir-199a	confirmed
2	hsa-mir-155	confirmed	27	hsa-mir-19b	confirmed
3	hsa-mir-146a	confirmed	28	hsa-mir-9	confirmed
4	hsa-mir-221	confirmed	29	hsa-mir-34c	confirmed
5	hsa-mir-29a	confirmed	30	hsa-mir-141	confirmed
6	hsa-mir-143	confirmed	31	hsa-let-7i	confirmed
7	hsa-mir-125b	confirmed	32	hsa-mir-200a	confirmed
8	hsa-mir-20a	confirmed	33	hsa-mir-15a	confirmed
9	hsa-mir-34a	confirmed	34	hsa-mir-101	confirmed
10	hsa-let-7a	confirmed	35	hsa-mir-28	confirmed
11	hsa-mir-133a	confirmed	36	hsa-mir-34b	confirmed
12	hsa-mir-15b	confirmed	37	hsa-mir-142	confirmed
13	hsa-mir-29b	confirmed	38	hsa-mir-196a	confirmed
14	hsa-mir-1	confirmed	39	hsa-mir-192	confirmed
15	hsa-mir-132	confirmed	40	hsa-mir-133b	confirmed
16	hsa-mir-30b	confirmed	41	hsa-mir-206	confirmed
17	hsa-let-23b	confirmed	42	hsa-mir-31	confirmed
18	hsa-let-7c	confirmed	43	hsa-let-7d	confirmed
19	hsa-mir-223	confirmed	44	hsa-mir-20b	confirmed
20	hsa-mir-375	confirmed	45	hsa-mir-137	confirmed
21	hsa-let-7f	confirmed	46	hsa-mir-29c	confirmed
22	hsa-mir-19a	confirmed	47	hsa-mir-181b	confirmed
23	hsa-mir-16	confirmed	48	hsa-mir-7	confirmed
24	hsa-mir-107	confirmed	49	hsa-mir-148a	confirmed
25	hsa-mir-222	confirmed	50	hsa-mir-30a	confirmed

**Table 6:** Top 50 esophageal neoplasms-related miRNAs predicted by MGCNSS

RANK	miRNA name	Evidence	RANK	miRNA name	Evidence
1	hsa-mir-125b	confirmed	26	hsa-mir-10b	confirmed
2	hsa-mir-16	confirmed	27	hsa-let-7f	confirmed
3	hsa-mir-17	confirmed	28	hsa-mir-193b	confirmed
4	hsa-mir-9	confirmed	29	hsa-mir-132	confirmed
5	hsa-mir-221	confirmed	30	hsa-let-7d	confirmed
6	hsa-mir-195	confirmed	31	hsa-mir-124	confirmed
7	hsa-mir-222	confirmed	32	hsa-mir-30a	confirmed
8	hsa-mir-15b	confirmed	33	hsa-mir-302b	confirmed
9	hsa-mir-18a	confirmed	34	hsa-mir-103a	unconfirmed
10	hsa-mir-20b	confirmed	35	hsa-mir-194	confirmed
11	hsa-mir-7	confirmed	36	hsa-mir-137	confirmed
12	hsa-mir-29a	confirmed	37	hsa-mir-206	confirmed
13	hsa-mir-23b	confirmed	38	hsa-mir-181a	confirmed
14	hsa-mir-200b	confirmed	39	hsa-mir-95	confirmed
15	hsa-mir-181b	confirmed	40	hsa-mir-24	confirmed
16	hsa-mir-142	confirmed	41	hsa-mir-218	confirmed
17	hsa-let-7i	confirmed	42	hsa-mir-30c	confirmed
18	hsa-mir-107	confirmed	43	hsa-mir-32	confirmed
19	hsa-mir-106a	confirmed	44	hsa-mir-429	confirmed
20	hsa-mir-1	confirmed	45	hsa-mir-29b	confirmed
21	hsa-mir-302c	confirmed	46	hsa-mir-302d	confirmed
22	hsa-mir-19b	confirmed	47	hsa-mir-372	confirmed
23	hsa-mir-133b	confirmed	48	hsa-mir-144	confirmed
24	hsa-mir-106b	confirmed	49	hsa-mir-128	confirmed
25	hsa-mir-146b	confirmed	50	hsa-mir-191	confirmed

Besides, to verify the ability of MGCNSS in finding novel miRNA–disease associations, we select Colon neoplasms and predict its top-10 associated miRNAs. Meanwhile, we also select part of the baseline approaches in Table 1 and predict their top-10 associated miRNAs of Colon neoplasms and sort their ranks according to their scores. The corresponding results are displayed in Table 7. The results demonstrate that all the top-10 predicted miRNAs by MGCNSS could be confirmed by HMDDv4.0 [63] and dbDEMC [64] database. In particular, hsa-Let-7a could be predicted by MGCNSS (Rank 10), while the remained approaches could not infer that this miRNA has an association relationship with Colon neoplasms in the top-10 predicted results. The miRNA hsa-Let-7a has been confirmed by PMID-31434447 in the HMDD database and SourceID-GSE2564 in dbDEMC. The results of this experiment could illustrate the stable prediction ability of the proposed model.

**Table 7:** Top 10 colon neoplasms-related miRNAs predicted by MGCNSS and other baseline approaches

Methods	Rank1	Rank2	Rank3	Rank4	Rank5	Rank6	Rank7	Rank8	Rank9	Rank10
MGCNSS	hsa-mir-21	hsa-mir-155	hsa-mir-146a	hsa-mir-221	hsa-mir-29a	hsa-mir-143	hsa-mir-125b	hsa-mir-20a	hsa-mir-34a	hsa-Let-7a
SVM [18]	hsa-mir-21	hsa-mir-181a	hsa-mir-183	hsa-mir-210	hsa-mir-208a	hsa-mir-146a	hsa-mir-34a	hsa-mir-31	hsa-mir-137	hsa-mir-30
RF [53]	hsa-mir-223	hsa-mir-126	hsa-mir-21	hsa-mir-146a	hsa-mir-145	hsa-mir-107	hsa-mir-210	hsa-mir-103	hsa-mir-486	hsa-mir-99a
XGBoost [54]	hsa-mir-21	hsa-mir-155	hsa-mir-34c	hsa-mir-181a	hsa-mir-221	hsa-mir-16	hsa-mir-200c	hsa-mir-133b	hsa-mir-182	hsa-mir-449b
GCN [55]	hsa-mir-132	hsa-mir-34c	hsa-mir-20a	hsa-mir-223	hsa-mir-19a	hsa-mir-192	hsa-mir-199a	hsa-mir-214	hsa-let-7f	hsa-mir-155
GAT [56]	hsa-mir-143	hsa-mir-29a	hsa-mir-16	hsa-mir-133b	hsa-mir-19b	hsa-mir-15b	hsa-mir-107	hsa-mir-141	hsa-mir-30a	hsa-mir-31
DTIGAT [57]	hsa-mir-133a	hsa-mir-20a	hsa-mir-29b	hsa-mir-125b	hsa-mir-155	hsa-mir-181a	hsa-mir-106b	hsa-mir-15b	hsa-mir-127	hsa-mir-137
DTICNN [3]	hsa-mir-15b	hsa-mir-125b	hsa-mir-19b	hsa-mir-34a	hsa-mir-155	hsa-mir-107	hsa-mir-7	hsa-mir-21	hsa-mir-200a	hsa-mir-34c
NeoDTI [35]	hsa-mir-155	hsa-mir-221	hsa-mir-15b	hsa-mir-30b	hsa-mir-223	hsa-mir-28	hsa-let-7i	hsa-mir-1	hsa-mir-21	hsa-mir-143
AMHMDA [13]	hsa-mir-17	hsa-mir-221	hsa-mir-20a	hsa-mir-34a	hsa-mir-155	hsa-mir-19a	hsa-mir-148a	hsa-mir-222	hsa-mir-21	hsa-mir-101

Moreover, to comprehensively investigate and compare the ability of MGCNSS to find novel associations, we conduct this experiment. Specifically, we choose Colon neoplasms as the target disease and collect its corresponding associated miRNAs by each comparison model with their predicted scores. All the predicted miRNAs for the comparison model will form their corresponding predicted miRNA set. Without loss of generality, we name the predicted miRNA set of MGCNSS as $A$, and the predicted miRNA set of each comparison approach as $B$. Then, we evaluate the results of MGCNSS with each baseline one by one under three metrics of $A$ and $B$, which are $|A \cap B| /|A \cup B|$, $|A|/|A \cup B|$ and $|B| / |A \cup B|$, respectively. The results are displayed in Table 8. Specifically, the values in column $|A|/|A \cup B|$ are always larger than those in column $|B| / |A \cup B|$. Taking MGCNSS and GATMDA for example, the value for $|A|/|A \cup B|$ is 0.9164, while the value for $|B|/|A \cup B|$ is 0.6104. From the results, we can find that MGCNSS outperforms other baselines in finding novel miRNA–disease associations.

**Table 8:** The comparison results of MGCNSS and other baselines in finding novel associations

Conbinations	${\rm |A\cap B|/|A \cup B|}$	${\rm |A|/|A \cup B|}$	${\rm |B| / |A \cup B|}$
MGCNSS vs SVM [18]	0.5864	0.8951	0.6914
MGCNSS vs RF [53]	0.6387	0.9355	0.7032
MGCNSS vs XGBoost [54]	0.7338	0.9416	0.7922
MGCNSS vs GCN [55]	0.3636	0.8788	0.4848
MGCNSS vs GAT [56]	0.3000	0.8529	0.4471
MGCNSS vs DTIGAT [57]	0.3016	0.7672	0.5344
MGCNSS vs DTICNN [58]	0.4304	0.9177	0.5127
MGCNSS vs NeoDTI [59]	0.3436	0.8896	0.4540
MGCNSS vs MSGCL [3]	0.2970	0.8788	0.4182
MGCNSS vs AMHMDA [35]	0.3980	0.8011	0.5967
MGCNSS vs GATMDA [13]	0.5519	0.9146	0.6104

## DISCUSSIONS

MGCNSS achieves the best performance among all the 11 miRNA–disease association prediction approaches. Extensive results could fully demonstrate its effectiveness and stability in different conditions. In this section, we would like to analyze the advantages and drawbacks as follows.

### The meta-path based multi-layer graph convolution

In the miRNA–disease association network, there are two types of nodes and two types of edges. The meta-paths with different lengths have rich semantic meaning between nodes in this heterogeneous network. For example, meta-path miRNA1-miRNA2-disease1 denotes that if miRNA1 and miRNA2 have a higher similar value, and miRNA2 and disease1 have an association relationship, miRNA1 may also have an association relationship with disease1 with high probability. This assumption has been widely accepted and utilized [8].

MGCNSS adopts the multi-layer graph convolution could capture the meta-paths with different lengths. It could comprehensively learn the embeddings of miRNAs and diseases, which could improve the prediction performance. The results of the ablation experiments demonstrate that the meta-based multi-layer graph convolution is essential for MGCNSS (see Figures 5 and 6). Besides, the performance of MGCNSS based on meta-paths with different lengths (See Table 3) is also well investigated. The results illustrate that meta-paths with length 1 and 2 have the best performance. The meta-path with length 3 may introduce noise, which lowers the indicators of MGCNSS in the experiment. In the next work, we would like to analyze the effect of network quality on the meta-path-based embedding learning.

### Negative sample selection strategy

According to the ablation study, we can find that the negative sample selection strategy has a great impact on the performance of MGCNSS. To verify the effectiveness of MGCNSS, we conduct the ablation study. The results show that the proposed negative sample selection strategy is of great help in improving the performance of MGCNSS. To visually demonstrate the role of negative sample selection, we depict two sub-figures in Figure 12, which indicates that with the increase of epoch number, the boundary between the positive and negative pairs is gradually clear. Besides, Figure 13 presents that MGCNSS could avoid selecting the negative samples that belong to the positive sample gather area (see Figure 13B). Our intuition is that samples in belong to the positive sample gather area should be the likely positive samples, not the likely negative samples. In this way, MGCNSS could select the more high-quality negative samples and have a better prediction result.

### The result of case study

It is crucial to discover novel association relationships between miRNAs and diseases for each prediction model. To verify this ability of MGCNSS, we conduct this case study. In the first group experiment, MGCNSS is employed to infer miRNAs for disease colon neoplasm and esophageal neoplasm respectively. Results demonstrate that the proposed approach has a creditable performance. All the top-50 predicted miRNAs for colon neoplasms and 49 of the top 50 for esophageal neoplasm could be verified by HMDD or dbDEMC.

Besides, we also present the top-10 predicted miRNAs for colon neoplasm by MGCNSS as well as other baseline approaches. The results demonstrate that all the top-10 predicted miRNAs by MGCNSS are identified in HMDD or dbDEMC. More importantly, for miRNA hsa-let-7a, MGCNSS could infer that it has an association relationship with colon neoplasm, while the other baseline approaches could not find this association relationship. Therefore, MGCNSS has a more powerful ability to find novel associations.

## CONCLUSION

In this study, we proposed MGCNSS for miRNA–disease association prediction based on a multi-layer graph convolution and negative sample selection strategy. Specifically, MGCNSS employs multi-layer graph convolution to automatically capture the meta-path relations with different lengths in the heterogeneous network and learn the discriminative representations of miRNAs and diseases. Besides, MGCNSS establishes a high-quality negative sample set by choosing the likely negative samples from the unlabeled sample set with the distanced-based sample selection strategy. The extensive results fully demonstrate that MGCNSS outperforms all baseline methods on the experimental dataset under different scenarios. The results of the case study further demonstrate the effectiveness of MGCNSS in miRNA–disease association prediction.

We will perform future works from the following three aspects. Firstly, some other biological entity association information such as miRNA-lncRNA associations could be employed for measuring the similarities of miRNAs and diseases from more comprehensive perspectives. In this way, a high-quality miRNA–disease heterogeneous network could be established, enabling learning more discriminative embeddings of miRNAs and drugs. Secondly, we could construct the miRNA–disease-related-biological knowledge graph, and predict the underlying associations between miRNA–disease by employing the knowledge graph embedding technique. Thirdly, since the association relationship prediction problem between different entities is one of the foundation tasks in bioinformatics, we would like to try to apply our proposed model to other link prediction problems, such as the disease–gene association, and microbe–drug association prediction task.

**Key Points**

- MGCNSS could automatically capture the rich semantics of meta-path with different lengths between miRNAs and diseases for learning their embeddings.
- A negative sample selection strategy is proposed to screen out high-quality negative samples to enhance the performance of the prediction model.
- The results demonstrate that MGCNSS outperforms all baseline methods on the evaluation metrics.

## Author Biographies

**Zhen Tian**, PhD (Harbin Institute of Technology), is an associate professor at the School of Computer and Artificial Intelligence, Zhengzhou University, Zhengzhou, China. His current research interests include computational biology, complex network analysis and data mining.

**Chenguang Han** is currently studying for a Master’s Degree of Computer Science and Technology at Zhengzhou University, Zhengzhou, China. His research interests include text mining, bioinformatics and deep learning.

**Lewen Xu** is studying for her Master’s degree of Software Engineering at Zhengzhou University, Zhengzhou, China. Her current research interests include computational biology, data mining and deep learning.

**Zhixia Teng** received a BS degree in information management and information systems from Northeast Forestry University in 2005, the MS and PhD degrees in computer science and technology from Northeast Forestry University in 2008, and from Harbin Institute of Technology in 2016, respectively. She is currently working at the College of Information and Computer Engineering, Northeast Forestry University. Her main research interests include machine learning and bioinformatics.

**Wei Song** received his PhD degree from Zhengzhou University, Zhengzhou China. He is an associate professor at the School of Computer and Artificial Intelligence, Zhengzhou University. His research interests include data science, data mining and machine learning.
